# Repeated colonization of alpine habitats by *Arabidopsis arenosa* viewed through freezing resistance and ice management strategies

**DOI:** 10.1111/plb.13454

**Published:** 2022-08-11

**Authors:** D. Kaplenig, C. Bertel, E. Arc, R. Villscheider, M. Ralser, F. Kolář, G. Wos, K. Hülber, I. Kranner, G. Neuner

**Affiliations:** ^1^ Department of Botany University of Innsbruck Innsbruck Austria; ^2^ Department of Botany Charles University of Prague Prague Czech Republic; ^3^ Department of Botany and Biodiversity Research University of Vienna Vienna Austria

**Keywords:** Adaptation, cold acclimation, freezing resistance, ice nucleation, parallel evolution, polyploidization

## Abstract

- Success or failure of plants to cope with freezing temperatures can critically influence plant distribution and adaptation to new habitats. Especially in alpine environments, frost is a likely major selective force driving adaptation. In *Arabidopsis arenosa* (L.) Lawalrée, alpine populations have evolved independently in different mountain ranges, enabling studying mechanisms of acclimation and adaptation to alpine environments.
- We tested for heritable, parallel differentiation in freezing resistance, cold acclimation potential and ice management strategies using eight alpine and eight foothill populations. Plants from three European mountain ranges (Niedere Tauern, Făgăraș and Tatra Mountains) were grown from seeds of tetraploid populations in four common gardens, together with diploid populations from the Tatra Mountains. Freezing resistance was assessed using controlled freezing treatments and measuring effective quantum yield of photosystem II, and ice management strategies by infrared video thermography and cryomicroscopy.
- The alpine ecotype had a higher cold acclimation potential than the foothill ecotype, whereby this differentiation was more pronounced in tetraploid than diploid populations. However, no ecotypic differentiation was found in one region (Făgăraș), where the ancient lineage had a different evolutionary history. Upon freezing, an ice lens within a lacuna between the palisade and spongy parenchyma tissues was formed by separation of leaf tissues, a mechanism not previously reported for herbaceous species.
- The dynamic adjustment of freezing resistance to temperature conditions may be particularly important in alpine environments characterized by large temperature fluctuations. Furthermore, the formation of an extracellular ice lens may be a useful strategy to avoid tissue damage during freezing.

## Graphical abstract

Testing for heritable, parallel differentiation in freezing resistance and ice management strategies, this study shows that alpine *Arabidopsis arenosa* populations, which evolved independently in different mountain ranges, have a higher potential to cold acclimate as compared to foothill populations.

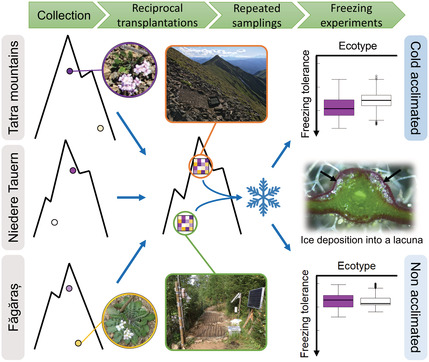

## INTRODUCTION

Temperature is one of the most prominent environmental factors that changes along elevation gradients (Körner 2003). Compared to lowland habitats, high elevation environments are characterized by lower night temperatures, higher daily temperature amplitudes, a longer duration of snow cover and more frequent and severe frost events (Taschler & Neuner 2004; Ladinig *et al*. 2013; Neuner 2014). Frost, especially when occurring during the vegetation period, has been considered a major factor that plants must be able to cope with in alpine environments, and a major evolutionary force driving adaptation to alpine environments (Körner 2003).

Plant response to environmental conditions includes acclimation, defined here as adjustments in form and function of an individual plant to its growth environment (Stearns & Hoekstra 2005). The capacity to acclimate is constrained by an individual's genetic constitution within a given range of phenotypic plasticity (Pfennig *et al*. 2010), *i.e*. the ability of a genotype/population to produce different phenotypes in response to different environmental conditions. In the long term, specific traits that contribute to higher fitness in a given environment may be favoured and maintained by natural selection, and can become heritably manifest as evolutionary adaptation (Stearns & Hoekstra 2005), defined here as heritable adjustments to environmental factors in which a plant evolved. In the course of evolution, adaptation to local environmental conditions can lead to the formation of ecotypes, these are locally adapted (groups of) usually still inter‐fertile, but morphologically and physiologically distinct populations within one species (Turesson 1922; Clausen *et al*. 1940; Lowry 2012). Due to their close genetic relationship but relatively high degree of differentiation in their adaptive traits, ecotypes have received much interest for over a century, as reviewed in Lowry (2012).

The availability of ecotypes resulting from parallel evolution, *i.e*. the independent evolution of the same traits in closely related lineages (Schluter *et al*. 2004), offers unique opportunities for addressing questions on evolutionary drivers of adaptation to distinct environments. Whereas parallel evolution has been extensively studied in animals, reports on plants are rare. Examples include *Argyranthemum sundingii* L. Borgen (Brochmann *et al*. 2000), *Cerastium alpinum* L. (Berglund *et al*. 2004), *Eucalyptus globulus* Labill. (Foster *et al*. 2007), *Senecio lautus* G.Forst. ex Willd. (Roda *et al*. 2013), *Silene vulgaris* (Moench) Garcke (Schat *et al*. 1996), *Heliosperma pusillum* Waldst. & Kit. (Trucchi *et al*. 2017) and *Arabidopsis arenosa* (L.) Lawalrée, an emerging new model plant (Knotek *et al*. 2020) suitable to study traits involved in the adaptation of herbaceous plants to the alpine environment, such as freezing resistance. *Arabidopsis arenosa* comprises diploid and autotetraploid populations occurring within a large distribution range from Southwest to Northern Europe, occupying a broad ecological amplitude from dry steppes and shaded rocks to coastal sand dunes and high‐alpine screes (Kolář *et al*. 2016a; Kolář *et al*. 2016b). However, a morphologically distinct alpine ecotype occupying elevations between ~1500 and 2500 m a.s.l. evolved independently in different mountain ranges, as recently shown by the genetic clustering inferred from genome‐wide single nucleotide polymorphism (SNP) markers (Knotek *et al*. 2020) and using coalescent simulations (Bohutínská *et al*. 2021). Individuals of the alpine ecotype are typically smaller, have fewer inflorescence stalks bearing less, but bigger, white or pinkish flowers (see Knotek *et al*. 2020 and Bohutínská *et al*. 2021, for representative pictures of both ecotypes). Plants of both ecotypes are perennial with slight differences in phenology. Individuals of the foothill ecotype usually flower earlier and for a longer period as compared to those of the alpine ecotype, which appears to have a stricter requirement for winter vernalisation. In addition, the alpine ecotype typically invests less into reproductive, but more into vegetative growth (Měsíček & Goliašová 2002, Wos *et al*. 2022).

Frost tolerant plants adjust their freezing resistance seasonally (Sakai & Larcher 1987; Gusta & Wisniewski 2013), with the highest resistance attained in the cold‐acclimated state in winter and the lowest in the non‐acclimated state during summer, with species‐specific differences (Taschler & Neuner 2004). Recent studies have demonstrated that freezing resistance is also adjusted in the non‐acclimated state during summer, and that repeated measurements of freezing resistance during the vegetation period may provide valuable insights into adaptation to habitats at different elevations (Bucher *et al*. 2018; Neuner *et al*. 2020). In general, seasonal changes in freezing resistance are mainly driven by temperature‐induced cold acclimation, de‐acclimation and re‐acclimation (Vyse *et al*. 2019). In herbaceous plants (in contrast to trees), day length is only of subsidiary importance to those processes (Sakai & Larcher 1987). Cold acclimation is induced by exposure to low but non‐freezing temperatures (2–6 °C) for a few days to several weeks (Xin & Browse 2000). In *A. thaliana*, cold acclimation can already be observed after 24 h at 4 °C, whereas full acclimation was only achieved after one week at 2 °C (Gilmour *et al*. 1988; Uemura *et al*. 1995). Moreover, exposure to sub‐zero temperatures can result in further increases in freezing resistance (Le *et al*. 2008). Conversely, de‐acclimation is generally a fast process lasting a few days (Pagter & Arora 2013; Zuther *et al*. 2015).

Despite this great potential to acclimate, heritable intraspecific differences in freezing resistance have been reported by Neuner *et al*. (2020, various species), Gianoli *et al*. (2004, *Colobanthus quietensis*), Hannah *et al*. (2006, *A. thaliana*), Zuther *et al*. (2012, *A. thaliana*), Li *et al*. (2005, *Salix paraplesia*), Melcher *et al*. (2000, *Metrosideros polymorpha*), Liu *et al*. (2019, *Rhododendron catawbiense*) and Armstrong *et al*. (2015, *Arabidopsis kamchatica*). A pivotal component of plant freezing resistance is the capability of controlling ice nucleation and ice propagation (Kuprian *et al*. 2014), which has been suggested to be particularly important for alpine plants (Hacker *et al*. 2011; Ladinig *et al*. 2013). Ice barriers within tissues can stop ice propagation into certain tissues, preventing or delaying ice growth, or as in *Ranunculus glacialis*, can be restricted to the spongy parenchyma tissue (Stegner *et al*. 2020a). Interestingly, heritable ecotypic differences in ice nucleation temperatures have been reported for herbaceous and woody species (Melcher *et al*. 2000; Hoermiller *et al*. 2018). However, it is unknown how leaves of *Arabidopsis* sp. freeze and where ice forms in the leaves.

Most studies into freezing resistance so far have been conducted either under controlled environmental conditions in climate chambers or greenhouses, *e.g*. Zuther *et al*. (2012), or at natural growing sites but under different environmental conditions, *e.g*. Neuner *et al*. (2020), *i.e*. either under non‐natural or non‐comparable environmental conditions. These limitations could be overcome by reciprocal transplantation experiments using common gardens in environmentally contrasting natural habitats, allowing disentangling of genetic and environmental determinants of freezing resistance. The present paper reports on a common garden experiment at low and high elevation using eight foothill and eight alpine populations of *A. arenosa* from three mountain ranges, where parallel evolution of alpine ecotypes from foothill ecotypes occurred (Knotek *et al*. 2020). Two mountain ranges, Niedere Tauern (Austria) and Făgăraș (Romania), harbour only autotetraploid populations, whereas in the third range, the Tatra Mountains (Slovakia), genetically close diploid and autotetraploid populations exist, both of which were included in this study. The present study takes advantage of the background knowledge gained from extensive genetic and transcriptomic analyses in the *A. arenosa* model, using the same ecotypes and experimental design (Wos *et al*. 2019; Knotek *et al*. 2020; Bohutínská *et al*. 2021; Wos *et al*. 2021). These studies revealed that clearly differentiated alpine and foothill *A. arenosa* ecotypes exist as a result of parallel evolution, whereby alpine populations are more closely related to their foothill counterparts in their respective mountain ranges than to other alpine populations.

The following hypotheses were tested to elucidate whether populations of the alpine and the foothill ecotypes have a heritably different freezing resistance – a trait showing a highly acclimative response – and whether they differ in their potential for cold acclimation, defined as the process of gradual hardening leading to the development of freezing resistance in plants (Thomashow 1999). We hypothesized that, although populations of both ecotypes acquire freezing resistance in response to decreasing temperatures, alpine populations have a greater potential for cold acclimation than populations of foothill origin (ecotypic differentiation hypothesis). Furthermore, we tested whether an enhanced cold acclimation potential is repeatedly found in independently evolved alpine populations (parallel evolution hypothesis). In addition, taking advantage of the co‐occurrence of the diploid and tetraploid ecotypes from the Tatra Mountains, we tested if the capability for cold acclimation is affected by ploidy (ploidy hypothesis). Finally, we tested if ice nucleation temperatures differ between alpine and foothill populations and examined ice propagation patterns and ice management, which has received hardly any attention in the *Arabidopsis* genus.

## MATERIAL AND METHODS

### Plant material and common gardens

Plants were grown from seeds collected in 2014 from a minimum of ten mother plants of eight alpine and eight foothill *A. arenosa* populations from three  European mountain ranges, Niedere Tauern (Eastern Alps, Austria), Tatra Mountains (Western Carpathians, Slovakia) and Făgăraș (Southern Carpathians, Romania), all showing pronounced, comparable differentiation in environmental conditions between foothill and alpine habitats; see Table S1 and Knotek *et al*. (2020) for details. Seeds of two alpine and two foothill tetraploid populations were collected from each mountain range, in addition to two diploid foothill and alpine populations each from the Tatra Mountains.

The first generation of *A. arenosa* plants was grown under controlled conditions in growth chambers as described in Wos *et al*. (2021). Mature seeds were harvested and stored at 4 °C in darkness under dry conditions. In spring 2018, seeds of the second generation were stratified at 4 °C for 4 days prior to germination. Seedlings were transplanted into multipot‐trays, grown in a greenhouse under ambient light and temperature conditions with minimum temperatures above 8 °C, and transplanted into the four common gardens after they had developed at least four leaves.

Two common gardens were established within the natural habitats of foothill and alpine populations of *A. arenosa* in Styria, Austria (Niedere Tauern), one at 980 m a.s.l. in Aigen im Ennstal, and the other at 2320 m a.s.l. on the northern slope of Mt. Hohenwart (further details in Table S2 and Figure S1). In each common garden, competing plants were removed from the plots, and a minimum of 830 *A. arenosa* plants were randomly transplanted to 8–12 plots of 1 m^2^ each, with grids of 10 × 10 0.01 m^2^ cells in natural soil (Figure S1). Another set of common gardens was established in Tyrol at 610 m a.s.l. in the Botanical Garden of the University of Innsbruck (328 individuals) and at 1960 m a.s.l. in the Alpine Garden of the University of Innsbruck on Mt. Patscherkofel (320 individuals; further details in Table S2). In this experiment, individuals were potted into soil containing a mixture of “alpine soil” [consisting of leaf mould, topsoil, lavalit, peat, sand and rock meal (5:2:1:2:2:0.2) used routinely at the Botanical Garden Innsbruck], silicate‐sand and vermiculite (8:1:1). The pots were buried in sand and plants were watered when required. Individuals of both ecotypes and all regions were transplanted to all common gardens at time periods corresponding to the seedling emergence observed at the natural sites, which differed depending on elevation.

Microclimate data were recorded with climate stations (CR10X and CR1000; Campbell Scientific, Logan, USA) installed in each common garden. Measurements were taken at 6‐min intervals. Rosette leaf temperatures, as typically used for ecophysiological measurements (Cernusca, 1972), were recorded using Type‐T copper‐constant thermocouples placed on the lower leaf surfaces of 15 individuals, which were distributed over the entire area (Walton 1982; Lomas *et al*. 1971). The mean of leaf temperatures within the 6 days preceding sampling was calculated and is hereafter referred to as acclimation temperature and used as a reference to compare the freezing resistance of different populations, indicative of the leaves' thermal history. This timeframe was chosen based on previous studies demonstrating that freezing resistance of herbaceous plants is adjusted within a few days in response to temperature fluctuations (Uemura *et al*. 1995; Zuther *et al*. 2012; Pagter & Arora 2013; Zuther *et al*. 2015; Vyse *et al*. 2019; Takahashi *et al*. 2020). Additionally, the daily minimum leaf temperatures were determined and averages over the 6 days preceding each sampling were calculated.

### Freezing resistance

Leaves were sampled for freezing resistance assessment at dates before and after the winter snow coverage, at which plants were cold acclimated, and at time points during the growing season at which plants had not acclimated. This was intended to allow for comparison of freezing resistance in both non‐acclimated and cold‐acclimated plants, hence distinguishing ‘constitutive’ and ‘inducible’ differences in freezing resistance. For the various common gardens, different sampling dates were selected to look for recurring patterns in the freezing resistance differences between populations in plants that had experienced different environmental conditions. Fully developed and undamaged leaves from all surviving individuals were collected and pooled in sealable plastic vials (2.5 × 6 cm) at each of the six sampling dates, corresponding to different acclimation temperatures (Table S2). The number of leaves sampled per individual was adjusted to the minimum required to reach at least 56 and preferably 70 leaves per population, to minimize the impact of the sampling on plant performance. Hence, one to three leaves per individual were sampled in most instances. A few drops of tap water were added to prevent desiccation of the leaves, and samples were transported to the laboratory in a light‐protected cooling box at +5 °C.

Freezing treatments were conducted either immediately after sampling (for leaves collected in the Tyrolian common gardens), or within the next 28 h (for leaves transported to the lab from the Styrian common gardens). For each population, seven transparency sheets were prepared, to expose the leaves to six freezing temperature treatments and a control treatment, as described below. On each of the seven transparency sheets, eight to ten randomly selected leaves were taped (Transpore; 3M Austria, Vienna). When several leaves were collected from the same individual, they were systematically taped to different sheets to be tested at different temperatures. These sheets were immediately covered with a paper towel moistened with ice‐nucleation‐active (INA) bacterial suspension (*Pseudomonas syringae* van Hall 1902) to trigger ice nucleation at approximately −2 °C (Wisniewski *et al*. 2014). Sheets were placed into separate zip‐lock plastic bags and cooled down to six target temperatures (−3, −6, −9, −12, −15, −18 °C) at a rate of 3 K·h^−1^, which is close to naturally occurring cooling rates below 0 °C (Buchner & Neuner 2010), and kept for 4 h at the target temperatures. Then, samples were thawed at a rate of 3 K·h^−1^ until leaves had reached a final temperature of +5 °C (Neuner *et al*. 2020). As a control, leaves on one transparency sheet per population remained unfrozen at +5 °C.

Freezing injury to each leaf was assessed by maximum quantum yield of photosystem II (F_V_/F_M_) (Maxi‐Pam; Walz, Effeltrich, Germany) 5 days after the end of the freezing treatment as described in Neuner & Buchner (1999). LT_50_, defined as the temperature at which 50% of the tested leaves are considered lethally damaged, was determined fitting a Boltzmann function to the response curve of F_V_/F_M_ values against temperature in combination with bootstrapping according to Stegner *et al*. (2020a) with 100 repetitions (Table S3).

### Infrared differential thermal analysis (IDTA) and ice‐nucleation temperature

Patterns of ice propagation were investigated with an infrared camera using potted individuals (T650; Flir Systems, Danderyd, Sweden) from the Botanical Garden Innsbruck (n = 5 individuals per population) according to Kuprian *et al*. (2017) and Neuner & Lichtenberger (2020). IDTA allows visualizing the entire vasculature upon freezing and study of ice propagation patterns (Hacker & Neuner 2007; Hacker & Neuner 2008). IDTA experiments were conducted during early spring 2019. Rosette leaves were cooled in a temperature‐controlled freezer at a constant rate of 3 K·h^−1^ with the IR camera placed inside the freezer. Infrared measurements were taken at a frequency of 7.5 frames·s^−1^. During measurements, the pot was placed in a Styrofoam container, and a layer of foam rubber and cotton was inserted between the soil surface and the leaf rosettes for thermal insulation. To prevent freezing of the soil, pots were heated from below using a heating mat (ThermoLux; Witte + Sutor, Murrhardt, Germany). Soil temperature was precisely controlled during the experiment by using thermocouples placed into the soil: the heating mat was turned on when temperature dropped below 2.6 °C and off when soil temperature reached 5.6 °C, resembling natural conditions during transient frost events, *e.g*. during nights, when soil remains unfrozen and warmer than the air (Stegner *et al*. 2020). Infrared differential thermal analysis (IDTA) was applied, and data were analysed with the ResearchIR software (Flir Systems). By subtracting a reference image captured just before the onset of the freezing event from the subsequent frames during freezing, ice propagation can be monitored at high resolution (Hacker & Neuner 2008; Neuner & Lichtenberger 2020). For each measurement, type‐T copper‐constant thermocouples fixed to leaf surfaces were used to record leaf temperatures with a data logger (CR10; Campbell Scientific) every 2 s to determine freezing temperatures. The temperature measured by the thermocouples when the first freezing event was determined by IDTA was defined as the ice nucleation temperature.

### Cryomicroscopy

Leaves from plants of all populations (in total n = 35, 2–3 per population) grown in the common garden within the Botanical Garden Innsbruck were fixed in a specimen mount and observed with a reflected‐light microscope (BXFM‐F; Olympus Optical, Tokyo, Japan) placed inside the cooling compartment of a temperature‐controlled freezer (ProfiLine Taurus PLTA0987, National Lab, Germany) as described in Stegner *et al*. (2020b), enabling observation of leaves in the frozen state. The leaves were cooled at a rate of 3 K·h^−1^ down to −8 °C, and cross‐sections were made with a frozen razor blade. Pictures were taken immediately with a stills camera (UC90; Olympus Optical) mounted on the microscope. In addition, cross‐sections of leaves were made with a hand microtome (GLS 1; Schenkung Dapples, Switzerland) and observed with a light microscope (Olympus BX50, Olympus Optical) controlled by the software cellD (version 3.1).

### Statistical analysis

Linear mixed‐effects models (LME) implemented in the function ‘lmer’ of the package ‘lmerTest’ (Kuznetsova *et al*. 2017) were applied to compare freezing resistance among populations of *A. arenosa*, instead of simple linear regressions. LME allow accounting for two sources of dependence within the data, *i.e*. population of origin and common garden used as crossed random intercepts. A random factor ‘site’ was used to account for all variation related to the site (transplantation time and location) in all models, except for the comparison of ice nucleation temperatures, which were only measured on one site. One model was used to test for ecotypic differentiation in freezing resistance between foothill and alpine populations, and for parallel evolution of alpine populations in the three mountain ranges. Ecotype, acclimation temperature, mountain range and the interactions between ecotype–acclimation temperature and ecotype–mountain range were regressed as fixed effects on LT_50_ [LT_50_ ~ ecotype + T_mean_ + mountain range + ecotype: T_mean_ + ecotype: mountain range + (1¦site) + (1¦population)]. Parameters were estimated by optimizing the restricted maximum likelihood criterion. The same model structure, but replacing mountain range by ploidy, was used to test for differences between diploid and tetraploid populations from the Tatra Mountains [LT_50_ ~ ecotype + T_mean_ + ploidy + ecotype: T_mean_ + ecotype: ploidy + (1¦site) + (1¦population)]. Differences in ice nucleation temperatures were tested by using ice nucleation temperature as response and ecotype, mountain range and the interaction of ecotype and mountain range as fixed‐effects [INT ~ ecotype + mountain range + ecotype: mountain range + (1¦population) + (1¦T_mean_)]. Determination of ice nucleation was conducted in March and April 2019. To account for differences in temperatures preceding the days of ice nucleation measurements, the 6‐days mean of leaf temperature was calculated and considered as random factor in the model; population of origin was used as second random factor. The assumptions of normal distribution of residuals and random effects and of homogeneity of variances were checked. For each model, marginal (*R* ^2^_LMMm_) and conditional *R* ^2^ values (*R* ^2^_LMMc_) were determined using the function ‘r.squaredGLMM’ implemented in the package ‘MuMin’ (Burnham & Anderson 2002). All analyses were performed in R, with a significance threshold of 0.05 (R Core Team 2021).

## RESULTS

**Fig. 1:**
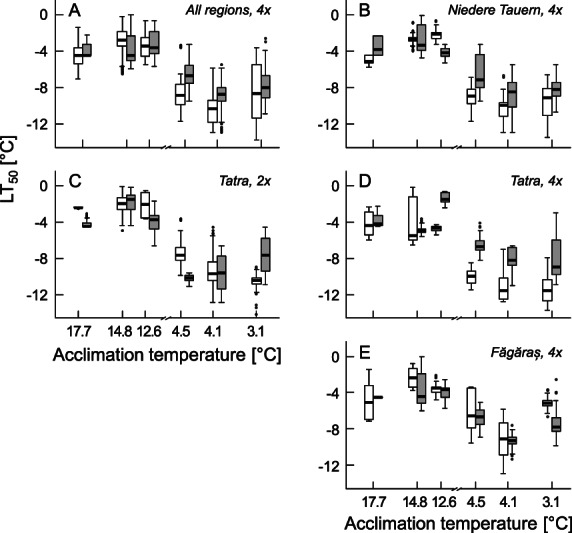
Cold acclimation of alpine and foothill populations originating from three mountain ranges. Freezing resistance of leaves of alpine (white) and foothill (grey) populations of *Arabidopsis arenosa* is expressed as LT_50_ (lower values indicate higher freezing resistance). (A) Comparison of all tetraploid (4x) alpine and all tetraploid foothill populations pooled from all mountain ranges. On the right is freezing resistance of tetraploid populations for each mountain range separately, *i.e*. (B) Niedere Tauern, (D) Tatra Mountains and (E) Făgăraș. (C) In the Tatra Mountains, diploid (2x) populations also occur, whose freezing resistance is shown for comparison with that of tetraploid populations. Box plots show medians and 25th and 75th percentiles. Dots outside 1.5× interquartile ranges represent outliers. Leaves were sampled from plants grown in common gardens on different sampling dates, and mean leaf temperatures averaged for a 6‐day period preceding the sampling were calculated from microclimate data recorded on‐site.

The combination of common gardens and sampling dates used allowed us to cover acclimation temperatures ranging from 3.1 to 17.7 °C (summarized in Table S2, together with the corresponding average daily minimum leaf temperatures). Overall, LT_50_ was lower at low acclimation temperature, reflecting an increase in freezing resistance (Fig. 1A), which was more pronounced in the alpine than the foothill ecotype, *i.e*. at ‘low’ acclimation temperatures (in the range between 3.1 and 4.5 °C), the alpine ecotype had lower LT_50_ values than the foothill ecotype, but not at the ‘higher’ temperatures (in the range between 12.6 and 17.7 °C). Considering the three mountain ranges separately, this pattern was similar for the Tatra Mountains (Fig. 1D) and the Niedere Tauern (Fig. 1B), but different for Făgăraș (Fig. 1E), where the alpine ecotype did not show a stronger decrease in LT_50_ with decreasing acclimation temperature compared to the foothill ecotype (Table 1). Hence, the different potential for cold acclimation of the alpine ecotype compared to the foothill ecotype was not consistently observed in plants originating from all three mountain ranges (Table 1).

**Table 1:** Freezing resistance of leaves of alpine and foothill tetraploid populations of *Arabidopsis arenosa.* Fixed effect coefficients of a linear mixed model relating ecotype, acclimation temperature (6‐day mean of leaf temperatures) and mountain range to freezing resistance, expressed as LT_50_. The alpine ecotype and the Tatra Mountains were used as baseline levels. Values in bold indicate significant (<0.05) marginal effects. Conditional (*R* ^2^_LMMm_) and marginal *R* ^2^ (*R* ^2^_LMMc_) of the model were 0.71 and 0.89, respectively.

	coefficient ± SE	*t*‐value	*P*‐value
Foothill ecotype	3.03 ± 0.66	4.62	**0.003**
Acclimation temperature	0.597 ± 0.01	106.67	**<0.001**
Niedere Tauern	1.14 ± 0.65	1.75	0.131
Făgăraș	2.45 ± 0.65	3.76	**0.009**
Foothill ecotype: Acclimation temperature	−0.01 ± 0.01	−15.20	**<0.001**
Foothill ecotype: Niedere Tauern	−1.32 ± 0.92	−1.43	0.204
Foothill ecotype: Făgăraș	−2.89 ± 0.92	−3.14	**0.020**

When diploid and tetraploid populations from the Tatra Mountains were compared, hardening to low temperatures was observed for both diploid and tetraploid populations and both ecotypes. However, tetraploid populations showed a stronger decrease in LT_50_ in response to decreasing temperature than diploid populations (Fig. 1C,D, Table 2). There were no clear differences in LT_50_ between diploid and tetraploid populations of the foothill ecotype, but tetraploid populations of the alpine ecotype had lower LT_50_ (higher freezing resistance) than their diploid counterparts after acclimation to falling temperatures (Fig. 1C,D). Differences in cold acclimation potential between ecotypes, as well as between diploid and tetraploid populations, were also found when the 6‐day mean of the daily minimum temperature was considered as acclimation temperature, instead of the 6‐days mean of leaf temperatures (Tables S4, S5).

**Table 2:** Freezing resistance of leaves of diploid and tetraploid populations of *Arabidopsis arenosa* originating from the Tatra Mountains. Fixed effect coefficients of a linear mixed model relating ecotype, acclimation temperature (6‐day mean of leaf temperatures) and ploidy to freezing resistance, expressed as LT_50_. The alpine ecotype and diploid ploidy level were used as baseline. Values in bold indicate significant (<0.05) marginal effects. Conditional (*R* ^2^_LMMm_) and marginal *R* ^2^ (*R* ^2^_LMMc_) of the model were 0.63 and 0.87, respectively.

	coefficient ± SE	*t‐*value	*P*‐value
Foothill ecotype	−0.59 ± 0.75	−0.79	0.476
Ploidy 4x	−1.19 ± 0.76	−1.57	0.191
Acclimation temperature	0.68 ± 0.01	100.34	**<0.001**
Ecotype Foothill: Ploidy 4x	2.72 ± 1.06	2.56	0.063
Ploidy 4x: Acclimation temperature	−0.08 ± 0.01	−10.62	**<0.001**

**Fig. 2:**
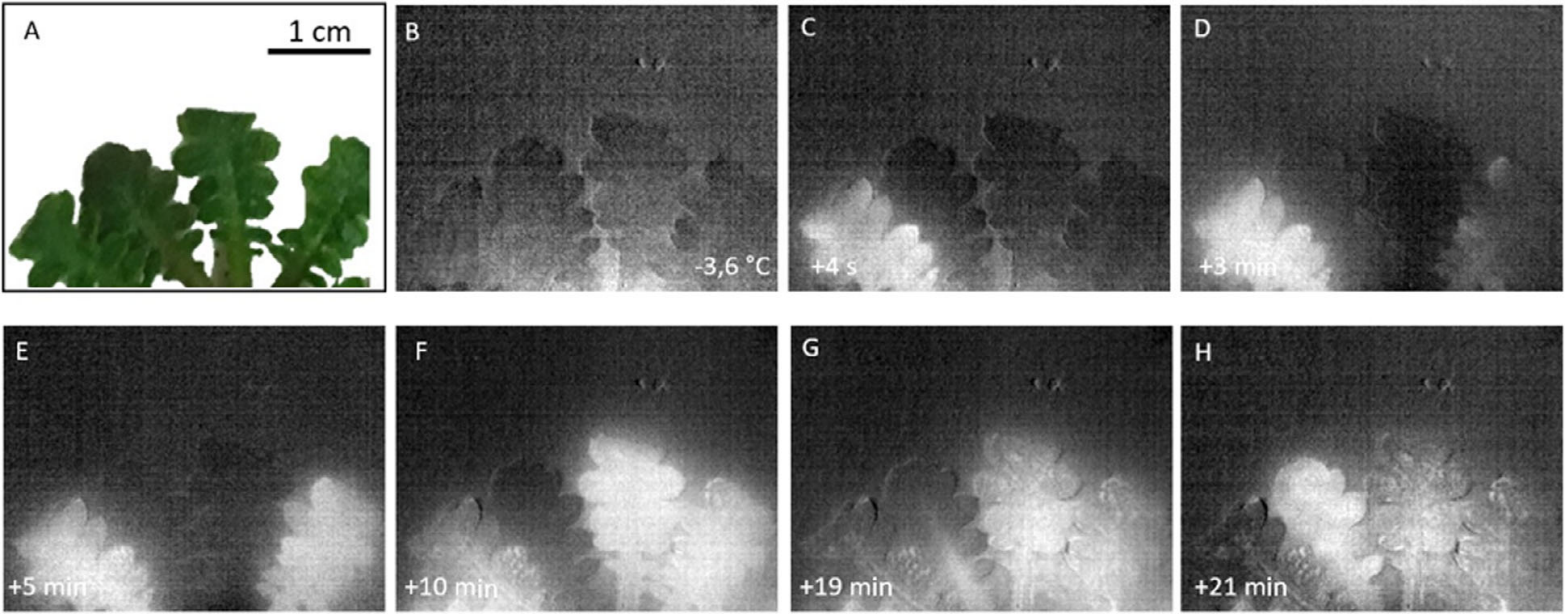
Ice formation and propagation in *Arabidopsis arenosa*, visualized using infrared differential thermal analysis (IDTA). (A) Part of rosette after exposure of the plant to a controlled freezing treatment. (B) Initiation of ice nucleation in the first leaf at −3.6 °C, triggered by INA bacteria at 10:29 h. (C–H) Time series of freezing pattern over 21 min. [Colour figure can be viewed at wileyonlinelibrary.com]

**Fig. 3:**
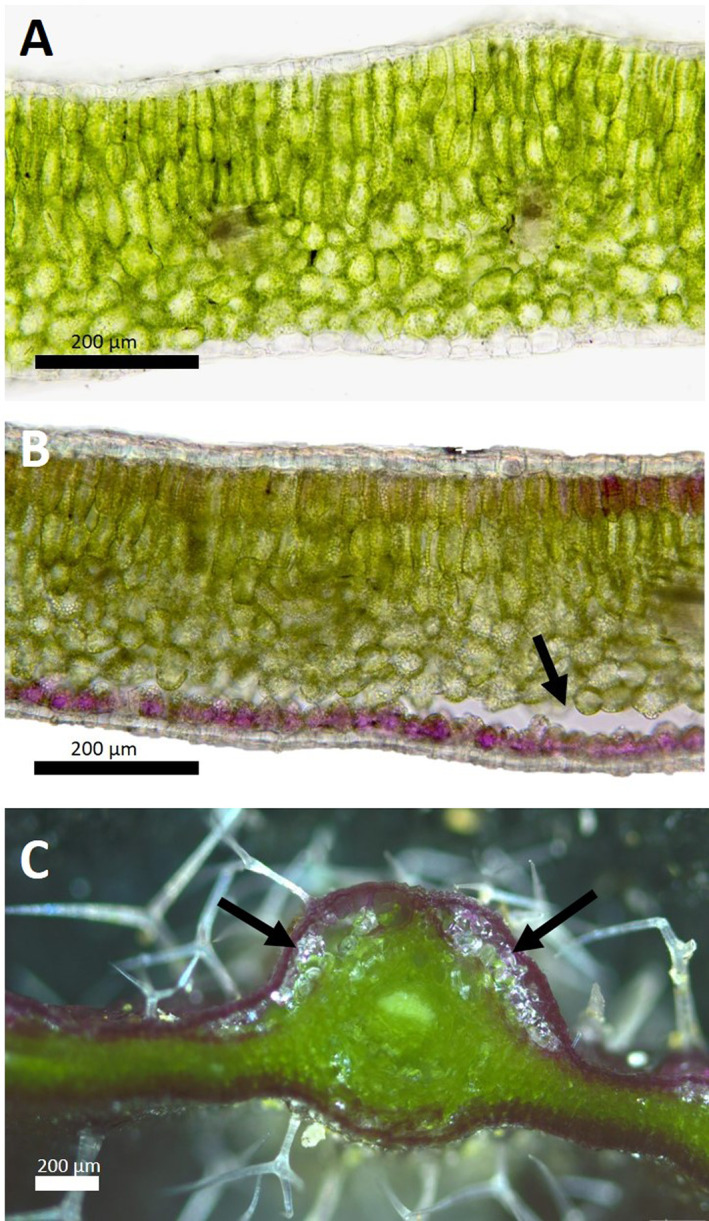
Extracellular ice formation in *Arabidopsis arenosa* leaves. Cross‐sections of (A) a current‐year leaf that was not exposed to freezing; (B) a leaf that had experienced freezing; (C) cross‐section with ice crystals in the lacuna, observed with cryomicroscopy. Arrows show the lacuna in the lower mesophyll, in which ice formed. [Colour figure can be viewed at wileyonlinelibrary.com]

The IDTA revealed a diffuse freezing pattern in the rosette leaves of *A. arenosa* lasting over 20 min (Fig. 2), with formation of large ice masses in the mesophyll of the leaves during freezing observed by light microscopy (Fig. 3). Either leaf rosettes immediately totally froze, or single leaves froze individually. When the leaves of *A. arenosa* were exposed to freezing, a large lacuna appeared in the mesophyll, giving space for ice growth. The formation of the lacuna dissociated the lower mesophyll from the lower epidermis, whereby one layer of mesophyll cells remained attached to the lower epidermis. In young, newly developed leaves that had not yet experienced natural freezing events, no lacuna was observed. Such ice propagation patterns and formation of a lacuna were observed in leaves of all populations and no differences between alpine and foothill populations or differences depending on the mountain ranges or ploidy were found. Ice nucleation temperatures did not differ significantly in leaves taken from plants of alpine populations, with no differences between populations from the three mountain ranges (Table 3, Figure S2A). In addition, ice nucleation temperatures did not differ between ecotypes of different ploidy level (Table 4, Figure S2B).

**Table 3:** Ice nucleation temperature of leaves of tetraploid populations of *Arabidopsis arenosa*. Fixed effect coefficients of a linear mixed model relating ecotype and mountain range to ice nucleation temperature. The alpine ecotype and the Tatra Mountains were used as baseline. Conditional (*R* ^2^_LMMm_) and marginal *R* ^2^ (*R* ^2^_LMMc_) of the model were 0.15 and 0.49, respectively.

	coefficient ± SE	*t‐*value	*P*‐value
Foothill ecotype	0.84 ± 0.73	1.15	0.295
Niedere Tauern	0.33 ± 0.73	0.45	0.670
Făgăraș	0.35 ± 0.75	0.47	0.653
Foothill ecotype: Niedere Tauern	0.01 ± 1.03	0.01	0.995
Foothill ecotype: Făgăraș	0.07 ± 1.04	0.07	0.947

**Table 4:** Ice nucleation temperature of leaves of diploid and tetraploid populations of *Arabidopsis arenosa* originating from the Tatra Mountains. Fixed effect coefficients of a linear mixed model relating ecotype and ploidy to ice nucleation temperature. The alpine ecotype and the Tatra Mountains were used as baseline. Conditional (*R* ^2^_GLMM(c)_) and marginal pseudo‐*R* ^2^ (*R* ^2^_GLMM(m)_) of the model were 0.11 and 0.49, respectively.

	coefficient ± SE	*t‐*value	*P*‐value
Ecotype Foothill	0.52 ± 1.05	0.49	0.648
Ploidy 4x	−0.22 ± 1.05	−0.21	0.845
Ecotype Foothill: Ploidy 4x	1.36 ± 1.49	0.91	0.412

## DISCUSSION

Plant survival and development in new habitats necessitate physiological acclimation, including cold acclimation in alpine habitats. The selection pressure imposed by the new environment can result in evolutionary adaptations (Stearns & Hoekstra 2005). The colonization of alpine habitats by *A. arenosa* led to parallel evolution of populations with a distinct alpine phenotype (Knotek *et al*. 2020). This close genetic relationship of ecologically divergent populations makes *A. arenosa* a good model to study freezing resistance, a key trait required to colonize cold environments (Preston & Sandve 2013). This paper contributes new knowledge of changes in cold acclimation potential during the course of parallel evolution in *A. arenosa*.

### Alpine ecotypes have a higher potential to acclimate to low temperatures than foothill ecotypes

The consistent increase in freezing resistance, viewed as LT_50_, in response to decreasing temperatures during the week preceding the freezing experiments showed that plants of both ecotypes and originating from all three mountain ranges were capable of cold acclimation (Fig. 1, Table 1). This finding supports a recent study showing that freezing resistance is adjusted during the vegetation period in various herbaceous species (Neuner *et al*. 2020). In *Arabidopsis thaliana*, cold acclimation can be acquired under short‐day and long‐day conditions (Zuther *et al*. 2012), whereas in woody plants, full acclimation requires a combination of low temperatures and short‐day conditions. Importantly, the alpine ecotype acclimated better to lower leaf temperatures, between 3.1 and 4.5 °C, than foothill ecotypes, demonstrating a clear, heritably determined difference between the two ecotypes (Fig. 1A), as shown by the significant interaction between ecotype and temperature (Table 1). Interestingly, for leaves that had experienced higher temperatures, *i.e*. acclimation temperatures between 12.6 and 17.7 °C, no such differences were observed between the alpine and the foothill ecotypes (Fig. 1A). A possible explanation for this finding is that the acquisition of constitutively high freezing resistance may not be advantageous as it comes with a metabolic cost. This may include the accumulation of cryo‐protective proteins and carbohydrates, and the suppression of photosynthesis and other metabolism‐related biochemical pathways for maintaining freezing resistance (Preston & Sandve 2013). Alternatively, selection for higher freezing resistance in alpine populations may have occurred only at low but not at high acclimation temperatures, typical of the alpine habitat.

As alpine plants experience wide amplitudes of temperature fluctuation and can also experience sub‐zero temperatures during the growing season (Körner 2003), dynamic adjustment of freezing resistance may be particularly important. In agreement with this assumption, differences in thermal acclimation potential were found between closely related ecotypes or populations of other plant species growing in alpine or polar environments. For example, in *Salix paraplesia*, an ecotype from higher latitudes acclimated better to decreasing temperatures than a southern ecotype (Li *et al*. 2005). Similarly, Antarctic ecotypes of *Colobanthus quietensis* had a higher acclimation potential than ecotypes from the Andes (Gianoli *et al*. 2004). Furthermore, populations of a mountain tree, *Polylepis australis*, from high elevations showed a more pronounced metabolic adjustment in response to cold than those from low elevations (Schrieber *et al*. 2020). Moreover, the acclimation potential of *A. thaliana* populations differed according to their geographic origin (Klotke *et al*. 2004). Taken together with these reports, data presented here suggest that the ability to cold acclimate during the growing season can evolve in closely related populations, driven by the variability in temperature in their respective habitats.

### The potential for cold acclimation evolved independently in geographically distinct alpine environments and is affected by ploidy

Although sharing a morphologically similar phenotype, the alpine populations included in the present study are more closely related to the foothill populations in their respective mountain ranges than to each other (Knotek *et al*. 2020). Alpine and foothill populations originating from the Niedere Tauern and the Tatra Mountains showed a similar differentiation in their cold acclimation potential, with alpine populations showing convergently a more pronounced reduction in LT_50_ at lower temperatures compared to foothill populations (Fig. 1B,D, Table 1). By contrast, no such clear differentiation in freezing resistance was observed for alpine and foothill populations from Făgăraș, where alpine populations did not have a lower LT_50_ at lower temperatures than foothill populations (Fig. 1E, Table 1a,b). This finding is in agreement with non‐parallel phenotypic differences in leaf traits in Făgăraș populations, which was interpreted as a result of a different evolutionary history of populations in this mountain range, despite the pronounced differences in environmental conditions between the foothill and the alpine habitat (Knotek *et al*. 2020). In the course of intraspecific parallel evolution, rapid adaptation often proceeds from sorting of ancestral standing genetic variation, rather than *de novo* mutations (Thompson *et al*. 2019), and the availability of standing adaptive variation (including freezing resistance candidate genes) scales with divergence in alpine *A. arenosa* (Bohutínská *et al*. 2021). Indeed, the Făgăraș populations represent the genetically most divergent lineage of the tetraploid *A. arenosa* complex range‐wide (Monnahan *et al*. 2019), which may explain why the cold acclimation potential of the alpine Făgăraș populations differed from those originating from the other two mountain ranges. Alternatively, differences in selection pressure in this mountain range could lead to the emergence of non‐parallel traits, but is assumed to be less likely due to similar environmental conditions across the alpine growing sites (Knotek *et al*. 2020). However, already small differences in selective environmental factors can greatly impact the phenotype, leading to non‐parallel patterns (Thompson *et al*. 2019).

We further studied if freezing resistance is influenced by ploidy using genetically closely related diploid and tetraploid populations from the Tatra Mountains (Wos *et al*. 2019). Polyploidisation events may lead to a higher adaptation potential (Soltis & Soltis 2016; Han *et al*. 2020; Arnold *et al*. 2015; Wos *et al*. 2019), especially under stressful conditions (Van de Peer *et al*. 2021; Novikova *et al*. 2020). Although the role of polyploidy is controversial in evolutionary theory, as early polyploids can face constraints, such as the regular segregation of additional chromosomes (Comai 2005), this is likely not the case for autotetraploid *A. arenosa*, which was shown to have stabilized meiosis and restored fertility (Yant *et al*. 2013). In accordance with the above‐mentioned hypothesis that polyploids have a higher adaptation potential, we found that freezing resistance in response to decreasing temperatures differed between ecotypes of tetraploid and diploid populations in the Tatra Mountains. However, these results contrast with recent studies from the same region, in which no ecological niche differentiation between the two *A. arenosa* cytotypes and overall phenotypic similarity at a given elevation was found (Wos *et al*. 2019; Morgan *et al*. 2020). Therefore, it seems likely that such an enhanced adaptation ability in polyploids, as found for the potential to cold acclimate, has not translated into niche expansion of tetraploids, at least within the environmental scale and region studied. However, an expansion of the ecological niche was found in tetraploid compared to diploid populations of *A. arenosa* across the whole distribution range, with tetraploids expanding towards boreal regions of Northern Europe (Molina‐Henao & Hopkins 2019), opening an area for further research.

### *Arabidopsis arenosa* shows an exceptional ice management strategy for herbaceous species

No differences between populations in experimentally induced ice nucleation temperatures was found (Table 3, Figure S2). Other authors have reported lower ice nucleation temperatures in populations from high elevations compared to low‐elevation populations of species occurring along an elevation gradient (*e.g. Metrosideros polymorpha*, Melcher *et al*. 2000). However, the ecological significance of ice nucleation temperatures determined in lab studies is debatable, as they often tend to overestimate the supercooling capability (Neuner & Hacker 2012). For instance, under high‐speed cooling at 1 °C·min^−1^, *A. thaliana* did not freeze at temperatures above −12°C (Rahman *et al*. 2021). Nevertheless, lower ice nucleation temperatures could be advantageous in habitats with frequent mild night frosts during the vegetation period, such as those at high elevation. It is not fully understood if extracellular ice formation and cellular freeze dehydration have negative after‐effects on physiological performance. If so, avoidance of extracellular freezing would be advantageous. However, in species inhabiting frost‐prone environments, such as *Senecio keniodendron* and *Lobelia telekii* (Bodner & Beck 1987) and *Ranunulus glacialis* (Stegner *et al*. 2020a), photosynthesis was not significantly affected subsequent to thawing, which may indicate little need for lowering of ice nucleation temperatures.

Furthermore, we studied ice management strategies using a combination of infrared video thermography and cryomicroscopy. In the leaves of herbaceous species, ice initially spreads through the vascular system, and IDTA allows visualization of the entire vasculature upon freezing (Hacker & Neuner 2007; Hacker & Neuner 2008). The ice management strategies did not differ between the ecotypes studied. In addition to ice nucleation temperature and ice management strategies, other processes contribute to frost tolerance and cold adaptation, such as cellular water content, osmotic regulation by different compounds (*e.g*. proline, betaine, polyols, sugars), antioxidant levels, cell wall modifications, membrane modifications, hormone balance and regulation of gene expression (Knight & Knight 2012). In the present study, IDTA revealed a scattered, diffuse freezing pattern in the rosette leaves of *A. arenosa* without initial freezing of the vascular system. Whether rosettes froze as a whole or sequentially did not depend on the population considered, but was likely influenced by the rosette architecture, as dense growth can provide thermal insulation to laminas and petioles, preventing the spread of ice from one leaf to another (Hacker *et al*. 2011). Interestingly, in single leaves, extracellular ice was also formed between the palisade and spongy parenchyma tissues, and the expanding ice masses led to the opening of a lacuna (Fig. 3). Similar IDTA freezing patterns were observed in the evergreen leaves of a woody plant, *Buxus sempervirens* (Hacker & Neuner 2007) that also formed an ice lens within a huge lacuna in the mesophyll upon freezing. In young *A. arenosa* leaves that had not yet been exposed to night frosts in nature, no lacuna was observed, indicating that the lacuna is formed at the first freezing event. In herbaceous species, ice lacunae were observed in petioles, but not in the lamina, of *Trifolium repens* and *Eschscholzia californica* leaves (McCully *et al*. 2004), and close to the vascular bundle of the petiole and adaxial leaf veins of the lamina of the perennial *Stachys byzantina* (Schott *et al*. 2020). The observation of the formation of an ice lacuna in the mesophyll is new for leaves of herbaceous species. Until now, ice was thought to be formed in intercellular spaces of herbaceous leaves. Here, we describe for the first time, that huge ice masses can grow in the leaves and form a lacuna. These ice masses would otherwise cause damage as they would not have enough space in the available intercellular spaces. Preliminary results showed that *A. thaliana* also forms an ice lacuna in the mesophyll.

In conclusion, we showed that cold acclimation is affected by ploidy and suggest that it has evolved independently in two mountain ranges. However, the absence of ecotypic differentiation in freezing resistance in the Făgăraș *A. arenosa* populations indicates that a heritable gain in cold acclimation potential was not required for the colonization of the alpine habitat in this particular mountain range. Whereas the two ecotypes did not differ in their ice management strategy, the formation of an ice lens within a lacuna between the palisade and spongy parenchyma tissues of the leaves was observed in all populations, a mechanism not previously reported for herbaceous species. Refinement and regulation of ice growth in lacunae is poorly studied, but appears to be a significant strategy to avoid tissue damage during freezing.

## CONFLICT OF INTEREST

The authors declare that the research was conducted in the absence of any commercial or financial relationships that could be construed as a potential conflict of interest.

## AUTHOR CONTRIBUTIONS

Material preparation and data collection were performed by DK, GN, EA, RV, MR and GW. CB and KH analysed the data. DK, CB, EA and IK wrote the manuscript, with significant inputs from all other authors. IK, GN, EA and FK conceived and designed the study. All authors read and approved the final manuscript.

## FUNDING INFORMATION

This study was funded by the Austrian Science fund FWF, grant P 31027 to Ilse Kranner.

## Supporting information


**Figure S1.** Common gardens used for reciprocal transplantation within the natural habitats of foothill and alpine *Arabidopsis arenosa* populations.
**Figure S2.** Ice nucleation temperatures determined in leaves of alpine and foothill populations of *Arabidopsis arenosa*.
**Table S1.** Population code, ecotype, mountain range, ploidy level, elevation and geographic coordinates (WGS 84) of the *Arabidopsis arenosa* populations of origin.
**Table S2.** Details of common garden locations, transplantation experiments and samplings for freezing resistance assessment.
**Table S4.** Freezing resistance of leaves of alpine and foothill, tetraploid populations of *Arabidopsis arenosa*.
**Table S5.** Freezing resistance of leaves of diploid and tetraploid populations of *Arabidopsis arenosa* originating from the Tatra mountains.

**Table S3.** Freezing resistance of leaves of alpine and foothill populations of *Arabidopsis arenosa*.

## Data Availability

The datasets generated for this study are available on request from the corresponding author.

## References

[plb13454-bib-0001] Armstrong J.J. , Takebayashi N. , Sformo T. , Wolf D.E. (2015) Cold tolerance in *Arabidopsis kamchatica* . American Journal of Botany, 102, 439–448. 25784477 10.3732/ajb.1400373

[plb13454-bib-0002] Arnold B. , Kim S.‐T. , Bomblies K. (2015) Single geographic origin of a widespread autotetraploid *Arabidopsis arenosa* lineage followed by interploidy admixture. Molecular Biology and Evolution, 32, 1382–1395. 25862142 10.1093/molbev/msv089

[plb13454-bib-0003] Berglund A.B.N. , Dahlgren S. , Westerbergh A. (2004) Evidence for parallel evolution and site‐specific selection of serpentine tolerance in *Cerastium alpinum* during the colonization of Scandinavia. New Phytologist, 161, 199–209.

[plb13454-bib-0004] Bodner M. , Beck E. (1987) Effect of supercooling and freezing on photosynthesis in freezing tolerant leaves of afro‐alpine ‘giant rosette’ plants. Oecologia, 72, 366–371. 28311131 10.1007/BF00377565

[plb13454-bib-0005] Bohutínská M. , Vlček J. , Yair S. , Laenen B. , Konečná V. , Fracassetti M. , Slotte T. , Kolář F. (2021) Genomic basis of parallel adaptation varies with divergence in *Arabidopsis* and its relatives. Proceedings of the National Academy of Sciences of the United States of America, 118, 2022713118. 10.1073/pnas.2022713118 PMC8166048 34001609

[plb13454-bib-0006] Brochmann C. , Borgen L. , Stabbetorp O.E. (2000) Multiple diploid hybrid speciation of the Canary Island endemic *Argyranthemum sundingii* (Asteraceae). Plant Systematics and Evolution, 220, 77–92.

[plb13454-bib-0007] Bucher S.F. , Feiler R. , Buchner O. , Neuner G. , Rosbakh S. , Leiterer M. , Römermann C. (2018) Temporal and spatial trade‐offs between resistance and performance traits in herbaceous plant species. Environmental and Experimental Botany, 157, 187–196.

[plb13454-bib-0008] Buchner O. , Neuner G. (2010) Freezing cytorrhysis and critical temperature thresholds for photosystem II in the peat moss *Sphagnum capillifolium* . Protoplasma, 243, 63–71. 19495938 10.1007/s00709-009-0053-8

[plb13454-bib-0009] Burnham K.P. , Anderson D.R. (2002) Model selection and multimodel inference: A practical information‐theoretic approach, 2nd edition. Springer, New York, USA.

[plb13454-bib-0010] Clausen J. , Keck D.D. , Hiesey W.M. (1940) Experimental studies on the nature of species. I. Effect of varied environments on western North American plants. Carnegie Institution of Washington, Washington, DC, USA.

[plb13454-bib-0011] Comai L. (2005) The advantages and disadvantages of being polyploid. Nature Reviews Genetics, 6, 836–846. 10.1038/nrg1711 16304599

[plb13454-bib-0012] Foster S.A. , McKinnon G.E. , Steane D.A. , Potts B.M. , Vaillancourt R.E. (2007) Parallel evolution of dwarf ecotypes in the forest tree *Eucalyptus globulus* . New Phytologist, 175, 370–380. 17587385 10.1111/j.1469-8137.2007.02077.x

[plb13454-bib-0013] Gianoli E. , Inostroza P. , Zuniga‐Feest A. , Reyes‐Diaz M. , Cavieres L.A. , Bravo L.A. , Corcuera L.J. (2004) Ecotypic differentiation in morphology and cold resistance in populations of *Colobanthus quitensis* (Caryophyllaceae) from the Andes of Central Chile and the maritime Antarctic. Arctic, Antarctic, and Alpine Research, 36, 484–489.

[plb13454-bib-0014] Gilmour S.J. , Hajela R.K. , Thomashow M.F. (1988) Cold‐acclimation in *Arabidopsis thaliana* . Plant Physiology, 87, 745–750. 16666219 10.1104/pp.87.3.745 PMC1054832

[plb13454-bib-0015] Gusta L.V. , Wisniewski M. (2013) Understanding plant cold hardiness: an opinion. Physiologia Plantarum, 147, 4–14. 22409670 10.1111/j.1399-3054.2012.01611.x

[plb13454-bib-0016] Hacker J. , Neuner G. (2007) Ice propagation in plants visualized at the tissue level by infrared differential thermal analysis (IDTA). Tree Physiology, 27, 1661–1670. 17938098 10.1093/treephys/27.12.1661

[plb13454-bib-0017] Hacker J. , Neuner G. (2008) Ice propagation in dehardened alpine plant species studied by infrared differential thermal analysis (IDTA). Arctic, Antarctic, and Alpine Research, 40, 660–670.

[plb13454-bib-0018] Hacker J. , Ladinig U. , Wagner J. , Neuner G. (2011) Inflorescences of alpine cushion plants freeze autonomously and may survive subzero temperatures by supercooling. Plant Science, 180, 149–156. 21151351 10.1016/j.plantsci.2010.07.013 PMC2987464

[plb13454-bib-0081] Han T.S. , Zheng Q.J. , Onstein R.E. , Rojas-Andrés B.M. , Hauenschild F. , Muellner-Riehl A.N. , Xing Y.W. (2020) Polyploidy promotes species diversification of Allium through ecological shifts. New Phytologist, 225, 571–583. 31394010 10.1111/nph.16098

[plb13454-bib-0019] Hannah M.A. , Wiese D. , Freund S. , Fiehn O. , Heyer A.G. , Hincha D.K. (2006) Natural genetic variation of freezing tolerance in *Arabidopsis* . Plant Physiology, 142, 98–112. 16844837 10.1104/pp.106.081141 PMC1557609

[plb13454-bib-0020] Hoermiller I.I. , Ruschhaupt M. , Heyer A.G. (2018) Mechanisms of frost resistance in *Arabidopsis thaliana* . Planta, 248, 827–835. 29936546 10.1007/s00425-018-2939-1

[plb13454-bib-0021] Klotke J. , Kopka J. , Gatzke N. , Heyer A.G. (2004) Impact of soluble sugar concentrations on the acquisition of freezing tolerance in accessions of *Arabidopsis thaliana* with contrasting cold adaptation – Evidence for a role of raffinose in cold acclimation. Plant, Cell & Environment, 27, 1395–1404.

[plb13454-bib-0022] Knight M.R. , Knight H. (2012) Low‐temperature perception leading to gene expression and cold tolerance in higher plants. New Phytologist, 195, 737–751. 22816520 10.1111/j.1469-8137.2012.04239.x

[plb13454-bib-0023] Knotek A. , Konečná V. , Wos G. , Požárová D. , Šrámková G. , Bohutínská M. , Zeisek V. , Marhold K. , Kolář F. (2020) Parallel alpine differentiation in *Arabidopsis arenosa* . Frontiers in Plant Science, 11, 561526. 33363550 10.3389/fpls.2020.561526 PMC7753741

[plb13454-bib-0024] Kolář F. , Fuxová G. , Záveská E. , Nagano A.J. , Hyklová L. , Lučanová M. , Kudoh H. , Marhold K. (2016a) Northern glacial refugia and altitudinal niche divergence shape genome‐wide differentiation in the emerging plant model *Arabidopsis arenosa* . Molecular Ecology, 25, 3929–3949. 27288974 10.1111/mec.13721

[plb13454-bib-0025] Kolář F. , Lucanova M. , Zaveska E. , Fuxova G. , Mandakova T. , Spaniel S. , Senko D. , Svitok M. , Kolnik M. , Gudzinskas Z. , Marhold K. (2016b) Ecological segregation does not drive the intricate parapatric distribution of diploid and tetraploid cytotypes of the *Arabidopsis arenosa* group (*Brassicaceae*). Biological Journal of the Linnean Society, 119, 673–688.

[plb13454-bib-0026] Körner C. (2003) Alpine plant life: functional plant ecology of High Mountain ecosystems, 2nd edition. Springer, Berlin, Germany.

[plb13454-bib-0027] Kuprian E. , Briceño V.F. , Wagner J. , Neuner G. (2014) Ice barriers promote supercooling and prevent frost injury in reproductive buds, flowers and fruits of alpine dwarf shrubs throughout the summer. Environmental and Experimental Botany, 106, 4–12. 25284910 10.1016/j.envexpbot.2014.01.011 PMC4104041

[plb13454-bib-0028] Kuprian E. , Munkler C. , Resnyak A. , Zimmermann S. , Tuong T.D. , Gierlinger N. , Müller T. , Livingston D.P. , Neuner G. (2017) Complex bud architecture and cell‐specific chemical patterns enable supercooling of *Picea abies* bud primordia. Plant, Cell & Environment, 40, 3101–3112. 10.1111/pce.13078 PMC5725666 28960368

[plb13454-bib-0029] Kuznetsova A. , Brockhoff P.B. , Christensen R.H. (2017) lmerTest package: Tests in linear mixed effects models. Journal of Statistical Software, 82, 1–26.

[plb13454-bib-0030] Ladinig U. , Hacker J. , Neuner G. , Wagner J. (2013) How endangered is sexual reproduction of high‐mountain plants by summer frosts? Frost resistance, frequency of frost events and risk assessment. Oecologia, 171, 743–760. 23386042 10.1007/s00442-012-2581-8 PMC3599211

[plb13454-bib-0031] Le M.Q. , Engelsberger W.R. , Hincha D.K. (2008) Natural genetic variation in acclimation capacity at sub‐zero temperatures after cold acclimation at 4 °C in different *Arabidopsis thaliana* accessions. Cryobiology, 57, 104–112. 18619434 10.1016/j.cryobiol.2008.06.004

[plb13454-bib-0032] Li C. , Wu N. , Liu S. (2005) Development of freezing tolerance in different altitudinal ecotypes of *Salix paraplesia* . Biologia Plantarum, 49, 65–71.

[plb13454-bib-0033] Liu B. , Xia Y.‐P. , Krebs S.L. , Medeiros J. , Arora R. (2019) Seasonal responses to cold and light stresses by two elevational ecotypes of *Rhododendron catawbiense*: a comparative study of overwintering strategies. Environmental and Experimental Botany, 163, 86–96.

[plb13454-bib-0034] Lomas J. , Schlesinger E. , Israeli A. (1971) Leaf temperature measurement techniques. Boundary‐Layer Meteorology, 1, 458–465.

[plb13454-bib-0035] Lowry D.B. (2012) Ecotypes and the controversy over stages in the formation of new species. Biological Journal of the Linnean Society, 106, 241–257.

[plb13454-bib-0036] McCully M.E. , Canny M. , Huang C. (2004) The management of extracellular ice by petioles of frost‐resistant herbaceous plants. Annals of Botany, 94, 665–674. 15355865 10.1093/aob/mch191 PMC4242212

[plb13454-bib-0037] Melcher P. , Cordell S. , Jones T. , Scowcroft P. , Niemczura W. , Giambelluca T. , Goldstein G. (2000) Supercooling capacity increases from sea level to tree line in the Hawaiian tree species *Metrosideros polymorpha* . International Journal of Plant Sciences, 161, 369–379. 10817972 10.1086/314271

[plb13454-bib-0038] Měsíček J. , Goliašová K. (2002) *Cardaminopsis* (C. A. Mey.) Hayek. In: Goliašová K. , Šípošová H. (Eds), Flóra Slovenska. Slovakia, Veda, Bratislava, pp 388–415.

[plb13454-bib-0039] Molina‐Henao Y.F. , Hopkins R. (2019) Autopolyploid lineage shows climatic niche expansion but not divergence in *Arabidopsis arenosa* . American Journal of Botany, 106, 61–70. 30609009 10.1002/ajb2.1212

[plb13454-bib-0079] Monnahan P. , Kolář F. , Baduel P. , Sailer C. , Koch J. , Horvath R. , Laenen B. , Schmickl R. , Paajanen P. , Šrámková G. , Bohutínská M. , Arnold B. , Weisman C.M. , Marhold K. , Slotte T. , Bomblies K. , Yant L. (2019) Pervasive population genomic consequences of genome duplication in *Arabidopsis arenosa* . Nature Ecology and Evolution, 3, 457–468. 30804518 10.1038/s41559-019-0807-4

[plb13454-bib-0040] Morgan E.J. , Čertner M. , Lučanová M. , Kubíková K. , Marhold K. , Kolář F. (2020) Niche similarity in diploid‐autotetraploid contact zones of *Arabidopsis arenosa* across spatial scales. American Journal of Botany, 107, 1375–1388. 32974906 10.1002/ajb2.1534

[plb13454-bib-0041] Neuner G. (2014) Frost resistance in alpine woody plants. Frontiers in Plant Science, 5, 654. 25520725 10.3389/fpls.2014.00654 PMC4249714

[plb13454-bib-0042] Neuner G. , Buchner O. (1999) Assessment of foliar frost damage: a comparison of i*n vivo* chlorophyll fluorescence with other viability tests. Angewandte Botanik, 73, 50–54.

[plb13454-bib-0043] Neuner G. , Hacker J. (2012) Ice formation and propagation in alpine plants, Plants in Alpine Regions. Springer, Vienna, Austria, pp 163–174.

[plb13454-bib-0044] Neuner G. , Lichtenberger E. (2020) Infrared thermal analysis of plant freezing processes. In: Hincha D.K. , Zuther E. (Eds), Plant cold acclimation. Springer, Cham, Switzerland, pp 33–41. 10.1007/978-1-0716-0660-5_4 32607973

[plb13454-bib-0045] Neuner G. , Huber B. , Plangger A. , Pohlin J.‐M. , Walde J. (2020) Low temperatures at higher elevations require plants to exhibit increased freezing resistance throughout the summer months. Environmental and Experimental Botany, 169, 103882.

[plb13454-bib-0046] Novikova P.Y. , Brennan I.G. , Booker W. , Mahony M. , Doughty P. , Lemmon A.R. , Lemmon E.M. , Roberts J.D. , Yant L. , Van de Peer Y. , Keogh J.S. , Donnellan S.C. (2020) Polyploidy breaks speciation barriers in Australian burrowing frogs *Neobatrachus* . PLoS Genetics, 16, e1008769. 32392206 10.1371/journal.pgen.1008769 PMC7259803

[plb13454-bib-0047] Pagter M. , Arora R. (2013) Winter survival and deacclimation of perennials under warming climate: Physiological perspectives. Physiologia Plantarum, 147, 75–87. 22583023 10.1111/j.1399-3054.2012.01650.x

[plb13454-bib-0048] Pfennig D.W. , Wund M.A. , Snell‐Rood E.C. , Cruickshank T. , Schlichting C.D. , Moczek A.P. (2010) Phenotypic plasticity's impacts on diversification and speciation. Trends in Ecology & Evolution, 25, 459–467. 20557976 10.1016/j.tree.2010.05.006

[plb13454-bib-0049] Preston J.C. , Sandve S.R. (2013) Adaptation to seasonality and the winter freeze. Frontiers in Plant Science, 4, 167. 23761798 10.3389/fpls.2013.00167 PMC3669742

[plb13454-bib-0050] Rahman T. , Shao M. , Pahari S. , Venglat P. , Soolanayakanahally R. , Qiu X. , Rahman A. , Tanino K. (2021) Dissecting the roles of cuticular wax in plant resistance to shoot dehydration and low‐temperature stress in *Arabidopsis* . International Journal of Molecular Sciences, 22, 1554. 33557073 10.3390/ijms22041554 PMC7913816

[plb13454-bib-0078] R Core Team . (2021) R: A language and environment for statistical computing. R Foundation for Statistical Computing, Vienna, Austria. Available from https://www.R-project.org/ (accessed 29 April 2022).

[plb13454-bib-0051] Roda F. , Ambrose L. , Walter G.M. , Liu H.L. , Schaul A. , Lowe A. , Pelser P.B. , Prentis P. , Rieseberg L.H. , Ortiz‐Barrientos D. (2013) Genomic evidence for the parallel evolution of coastal forms in the *Senecio lautus* complex. Molecular Ecology, 22, 2941–2952. 23710896 10.1111/mec.12311

[plb13454-bib-0052] Sakai A. , Larcher W. (1987) Low temperature and frost as environmental factors. In: Sakai A. , Larcher W. (Eds), Frost survival of plants. Springer, Berlin, Germany, pp 1–20.

[plb13454-bib-0053] Schat H. , Vooijs R. , Kuiper E. (1996) Identical major gene loci for heavy metal tolerances that have independently evolved in different local populations and subspecies of *Silene vulgaris* . Evolution, 50, 1888–1895. 28565612 10.1111/j.1558-5646.1996.tb03576.x

[plb13454-bib-0054] Schluter D. , Clifford E.A. , Nemethy M. , McKinnon J.S. (2004) Parallel evolution and inheritance of quantitative traits. The American Naturalist, 163, 809–822. 10.1086/383621 15266380

[plb13454-bib-0055] Schott R.T. , Neinhuis C. , Roth‐Nebelsick A. (2020) Extracellular ice formation in special intercellular air spaces in *Stachys byzantina* C. Koch. Feddes Repertorium, 131, 233–243.

[plb13454-bib-0056] Schrieber K. , Caceres Y. , Engelmann A. , Marcora P. , Renison D. , Hensen I. , Muller C. (2020) Elevational differentiation in metabolic cold stress responses of an endemic mountain tree. Environmental and Experimental Botany, 171, 103918.

[plb13454-bib-0080] Soltis P.S. , Soltis D.E. (2016) Ancient WGD events as drivers of key innovations in angiosperms. Current Opinion in Plant Biology, 30, 159–165. 27064530 10.1016/j.pbi.2016.03.015

[plb13454-bib-0057] Stearns S.C. , Hoekstra R.F. (2005) Evolution, an introduction, 2nd edition. Oxford University Press, Oxford, UK.

[plb13454-bib-0058] Stegner M. , Lackner B. , Schäfernolte T. , Buchner O. , Xiao N. , Gierlinger N. , Holzinger A. , Neuner G. (2020a) Winter nights during summer time: Stress physiological response to ice and the facilitation of freezing cytorrhysis by elastic cell wall components in the leaves of a nival species. International Journal of Molecular Sciences, 21, 7042. 32987913 10.3390/ijms21197042 PMC7582304

[plb13454-bib-0059] Stegner M. , Wagner J. , Neuner G. (2020b) Ice accommodation in plant tissues pinpointed by cryo‐microscopy in polarised light. Plant Methods, 16, 1–9. 32477423 10.1186/s13007-020-00617-1 PMC7240938

[plb13454-bib-0060] Takahashi D. , Zuther E. , Hincha D.K. (2020) Analysis of changes in plant cell wall composition and structure during cold acclimation. In: Hincha D. , Zuther E. (Eds), Plant cold acclimation. Methods in Molecular Biology. Humana, New York, USA. 10.1007/978-1-0716-0660-5_17 32607986

[plb13454-bib-0061] Taschler D. , Neuner G. (2004) Summer frost resistance and freezing patterns measured *in situ* in leaves of major alpine plant growth forms in relation to their upper distribution boundary. Plant, Cell & Environment, 27, 737–746.

[plb13454-bib-0062] Thomashow M.F. (1999) Plant cold acclimation: Freezing tolerance genes and regulatory mechanisms. Annual Review of Plant Biology, 50, 571–599. 10.1146/annurev.arplant.50.1.571 15012220

[plb13454-bib-0063] Thompson K.A. , Osmond M.M. , Schluter D. (2019) Parallel genetic evolution and speciation from standing variation. Evolution Letters, 3, 129–141. 31289688 10.1002/evl3.106 PMC6591551

[plb13454-bib-0064] Trucchi E. , Frajman B. , Haverkamp T.H. , Schönswetter P. , Paun O. (2017) Genomic analyses suggest parallel ecological divergence in *Heliosperma pusillum* (Caryophyllaceae). New Phytologist, 216, 267–278. 28782803 10.1111/nph.14722 PMC5601199

[plb13454-bib-0065] Turesson G. (1922) The species and the variety as ecological units. Hereditas, 3, 100–113.

[plb13454-bib-0066] Uemura M. , Joseph R.A. , Steponkus P.L. (1995) Cold acclimation of *Arabidopsis thaliana* (effect on plasma membrane lipid composition and freeze‐induced lesions*)* . Plant Physiology, 109, 15–30. 12228580 10.1104/pp.109.1.15 PMC157560

[plb13454-bib-0067] Van de Peer Y. , Ashman T.L. , Soltis P.S. , Soltis D.E. (2021) Polyploidy: an evolutionary and ecological force in stressful times. The Plant Cell, 33, 11–26. 33751096 10.1093/plcell/koaa015 PMC8136868

[plb13454-bib-0068] Vyse K. , Pagter M. , Zuther E. , Hincha D.K. (2019) Deacclimation after cold acclimation – a crucial, but widely neglected part of plant winter survival. Journal of Experimental Botany, 70, 4595–4604. 31087096 10.1093/jxb/erz229 PMC6760304

[plb13454-bib-0069] Walton W.H. (1982) Instruments for measuring biological microclimates for terrestrial habitats in polar and high alpine regions: a review. Arctic and Alpine Research, 14, 275–286.

[plb13454-bib-0070] Wisniewski M. , Gusta L. , Neuner G. (2014) Adaptive mechanisms of freeze avoidance in plants: a brief update. Environmental and Experimental Botany, 99, 133–140.

[plb13454-bib-0071] Wos G. , Mořkovská J. , Bohutínská M. , Šrámková G. , Knotek A. , Lučanová M. , Španiel S. , Marhold K. , Kolář F. (2019) Role of ploidy in colonization of alpine habitats in natural populations of *Arabidopsis arenosa* . Annals of Botany, 124, 255–268. 31185073 10.1093/aob/mcz070 PMC6758580

[plb13454-bib-0072] Wos G. , Bohutínská M. , Nosková J. , Mandáková T. , Kolář F. (2021) Parallelism in gene expression between foothill and alpine ecotypes in *Arabidopsis arenosa* . The Plant Journal, 105, 1211–1224. 33258160 10.1111/tpj.15105

[plb13454-bib-0073] Wos G. , Arc E. , Hülber K. , Konečná V. , Knotek A. , Požárová D. , Bertel C. , Kaplenig D. , Mandáková T. , Neuner G. , Schönswetter P. , Kranner I. , Kolář F. (2022) Parallel local adaptation to an alpine environment in *Arabidopsis arenosa* . Journal of Ecology, in press. 10.1111/1365-2745.13961

[plb13454-bib-0074] Xin Z. , Browse J. (2000) Cold comfort farm: the acclimation of plants to freezing temperatures. Plant, Cell & Environment, 23, 893–902.

[plb13454-bib-0075] Yant L. , Hollister J.D. , Wright K.M. , Arnold B.J. , Higgins J.D. , Franklin F.C.H. , Bomblies K. (2013) Meiotic adaptation to genome duplication in *Arabidopsis arenosa* . Current Biology, 23, 2151–2156. 24139735 10.1016/j.cub.2013.08.059 PMC3859316

[plb13454-bib-0076] Zuther E. , Schulz E. , Childs L.H. , Hincha D.K. (2012) Clinal variation in the non‐acclimated and cold‐acclimated freezing tolerance of *Arabidopsis thaliana* accessions. Plant, Cell & Environment, 35, 1860–1878. 10.1111/j.1365-3040.2012.02522.x 22512351

[plb13454-bib-0077] Zuther E. , Juszczak I. , Lee Y.P. , Baier M. , Hincha D.K. (2015) Time‐dependent de‐acclimation after cold acclimation in *Arabidopsis thaliana* accessions. Scientific Reports, 5, 12199. 26174584 10.1038/srep12199 PMC4648415

