# Supplementary material for: Repeated colonization of alpine habitats by Arabidopsis arenosa viewed through freezing resistance and ice management strategies
Source: Plant Biol (Stuttg). 2022 Aug 11;24(6):939–49. doi: 10.1111/plb.13454 (PMC9804731; doi:10.1111/plb.13454)
Supplement: Supplementary file 1 — Figure S1. Common gardens used for reciprocal transplantation within the natural habitats of foothill and alpine Arabidopsis arenosa populations. Figure S2. Ice nucleation temperatures determined in leaves of alpine and foothill populations of Arabidopsis arenosa. Table S1. Population code, ecotype, mountain range, ploidy level, elevation and geographic coordinates (WGS 84) of the Arabidopsis arenosa populations of origin. Table S2. Details of common garden locations, transplantation experiments and samplings for freezing resistance assessment. Table S4. Freezing resistance of leaves of alpine and foothill, tetraploid populations of Arabidopsis arenosa. Table S5. Freezing resistance of leaves of diploid and tetraploid populations of Arabidopsis arenosa originating from the Tatra mountains. [file PLB-24-939-s001.docx]

Supplementary Material

1. **Supplementary Figures**


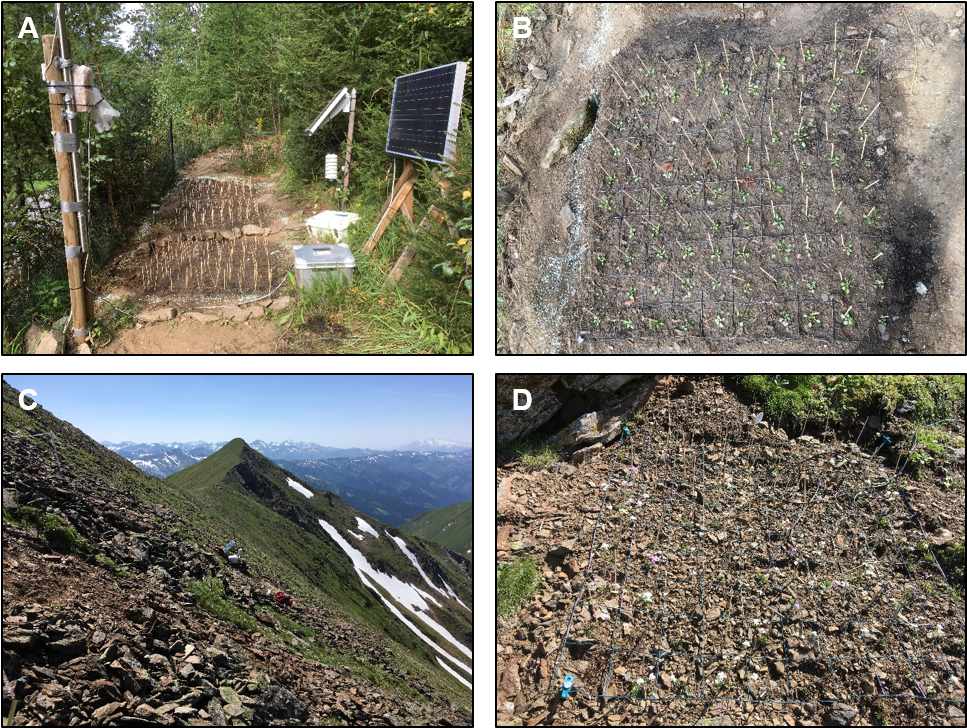


**Supplementary Figure 1. Common gardens used for reciprocal transplantation within the natural habitats of foothill and alpine *Arabidopsis arenosa* populations.** (A, C) Overview of the sites where the plots were set up in common gardens in Aigen im Ennstal (foothill site) and on Mt Hohenwart (alpine site), respectively. In Aigen im Ennstal, the plots were located at an altitude of 980 metres a.s.l. on the bench of a cutting slope above the road. On Mt Hohenwart, the plots were established at an altitude of 2297 metres a.s.l. on a rocky slope located on the north face just below the mountain top. On each site, seedlings were transplanted to 8 to 12 plots of 1 m^2^ each, each containing up to 100 plants. A climate station powered by solar panels was installed on each plot to monitor changes in temperature, irradiance and relative humidity, including thermocouples used to record leaf temperatures. (B, D) Representative pictures of plots set up at the foothill and alpine site, respectively. A metal grid was fixed to the ground to define the individual cells and seedlings from the 16 populations, which were transplanted to random positions within the plots, ensuring an equal distribution of the populations across all plots. An additional small wooden skewer was added next to the plants to ensure they could be distinguished from other seedlings that may have germinated from seeds in the soil seed bank.

**Supplementary Figure 2. Ice nucleation temperatures determined in leaves of alpine and foothill populations of *Arabidopsis arenosa.*** (A) Ice nucleation temperatures of tetraploid populations originating from three mountain ranges, i.e. the Tatra mountains (T), Niedere Tauern (NT) and Făgăraș (F). (B) Ice nucleation temperatures of diploid and tetraploid populations from the Tatra mountains. White and grey box plots show alpine and foothill populations, respectively. Box plots show medians and the 25^th^ and 75^th^ percentiles. Dots outside the 1.5 × interquartile ranges represent outliers. Leaves were sampled from plants grown in common gardens at different sampling dates and the acclimation temperatures, defined as the daily minimum temperature averaged for a six days period preceding the sampling, were calculated from microclimatic data recorded on each site.

1. **Supplementary Tables:**

Supplementary Table 1. Population code, ecotype, mountain range, ploidy level, elevation and geographic coordinates (WGS 84) of the *Arabidopsis arenosa* populations of origin. The population codes listed are the same as in previous studies using the same populations (Knotek *et al.* 2020, Wos *et al.* 2019, Wos *et al.* 2021); for comparability, the corresponding population reference (“Pop.”) in Bohutínská *et al.* (2021) is provided for the populations used in both in this study and in Bohutínská et al. (2021). Geographical maps displaying the habitats of origin of the different populations can be found in Knotek *et al.* (2020) and Bohutínská *et al.* (2021) along with representative pictures of the habitats and phenotypes of the alpine and foothill ecotypes. “A “denotes the alpine and “F” the foothill ecotype. VT: Vysoké Tatry, ZT: Západné Tatry, FG: Făgăraș, NT: Niedere Tauern.

| Code | Pop.^1^ | Lineage | Ecotype | Mountain range | Ploidy | Elevation of origin (m.a.s.l.) | Latitude (N) | Longitude (E) |
| --- | --- | --- | --- | --- | --- | --- | --- | --- |
| AA084 | VEL | VT | A | Tatra Mountains | 2x | 1,823 | 49.162 | 20.1542 |
| AA090 | ZEP | VT | A | Tatra Mountains | 2x | 1,625 | 49.2065 | 20.2151 |
| AA087 | - | ZT | A | Tatra Mountains | 4x | 2,031 | 49.197 | 19.7448 |
| AA168 | TKO | ZT | A | Tatra Mountains | 4x | 1,783 | 49.2045 | 19.7352 |
| AA065 | BAL | FG | A | Făgăraș Mountains | 4x | 2,269 | 45.602 | 24.6226 |
| AA222 | LAC | FG | A | Făgăraș Mountains | 4x | 2,092 | 45.5954 | 24.6346 |
| AA253 | SCH | NT | A | Niedere Tauern | 4x | 2,225 | 47.2777 | 14.3219 |
| AA254 | - | NT | A | Niedere Tauern | 4x | 2,360 | 47.3644 | 14.6808 |
| AA016 | SUB | VT | F | Tatra Mountains | 2x | 600 | 48.9603 | 20.3833 |
| AA208 | BAB | VT | F | Tatra Mountains | 2x | 844 | 49.0435 | 20.1808 |
| AA171 | HRA | ZT | F | Tatra Mountains | 4x | 720 | 49.0072 | 20.2864 |
| AA229 | - | ZT | F | Tatra Mountains | 4x | 673 | 49.2076 | 19.5494 |
| AA067 | DRA | FG | F | Făgăraș Mountains | 4x | 858 | 45.4416 | 25.2239 |
| AA251 | - | FG | F | Făgăraș Mountains | 4x | 915 | 45.4267 | 25.2133 |
| AA252 | - | NT | F | Niedere Tauern | 4x | 820 | 47.1826 | 14.3379 |
| AA255 | ING | NT | F | Niedere Tauern | 4x | 970 | 47.2842 | 14.6819 |

*^1^according to Bohutínská et al. (2021)*

Supplementary Table 2. Details of common garden locations, transplantation experiments and samplings for freezing resistance assessment. Seedlings from all *Arabidopsis arenosa* populations were transplanted to each of the four common gardens at a specified transplantation date (“TP date”) and later sampled for assessment of leaf freezing tolerance. For each of the sampling dates, the acclimation temperature was calculated as the average of the daily mean leaf temperature recorded during 6 days preceding sampling, and the mean of the daily minimum leaf temperature over the same period are indicated in brackets. The day length was calculated as the average of the length of 6 days preceding sampling. Number of transplanted individuals refers to individual plants that had survived one month after transplantation. The corresponding minimum and maximum numbers of individuals per population are indicated in brackets. Note that reduced numbers of individuals transplanted for some populations resulted from differences in seed availability, germination capacity and seedling establishment, and mostly concerned two out of the 16 populations for the first transplantations performed (AA087 and AA168).

| Site | Coordinates and elevation | TP date | Sampling date | Acclimation temperature | Day length [hours]^1^ | Number of transplanted individuals |
| --- | --- | --- | --- | --- | --- | --- |
| Aigen im Ennstal, Styria, Austria | 47°29'47.3"N 14°10'32.7"E  980 m a.s.l. | 09.05.2018  28.08.2019 | 14.10.2018  27.11.2019 | 12.6 ± 0.7°C (7.8 ± 1.2 °C)  4.5 ± 1.0°C (1.3 ± 1.4 °C) | 11.1 ± 0.3  8.2 ± 0.2 | 830 (25-66)  998 (61-63) |
| Mt. Hohenwart, Styria, Austria | 47°19'47.3"N 14°14'10.6"E  2,320 m a.s.l. | 01.07.2018 | 15.10.2018: | 3.1 ± 1.3 °C (-0.1 ± 1.3°C), | 10.4 ± 0.2 | 1044 (66-74) |
| Innsbruck Botanical Garden, Tyrol, Austria | 47°16'04.7"N 11°22'47.9"E  610 m a.s.l. | 12.06.2018 | 25.09.2018  18.03.2019 | 14.8 ± 2.3°C (9.8 ± 3.1°C)  4.1 ± 2.6 °C (0.0 ± 3.1 °C) | 12.2 ± 0.1  11.8 ± 0.1 | 328 (16-24) |
| Innsbruck,  Alpine Garden Mt. Patscherkofel, Tyrol, Austria | 47°12'38.8"N 11°27'05.7"E  1,960 m a.s.l. | 13.07.2018 | 17.06.2019 | 17.7 ± 5.1 °C (9.9 ± 5.9 °C) | 15.9 ± 0.0 | 320 (15-23) |

***^1^*** *For the common gardens in Styria, day length was determined from the climate data recorded at each site, defined as the duration during which PPFD was above 0 µmol m^-1^ s^-1^. For both common gardens established in Innsbruck, day length was calculated based on local sunrise and sunset data available from the ZAMG (Zentralanstalt für Meteorologie und Geodynamik, https://www.zamg.ac.at , accessed 11.04.2022).*

**Supplementary Table 4.** **Freezing resistance of leaves of alpine and foothill, tetraploid populations of *Arabidopsis arenosa.*** Fixed effect coefficients of a linear mixed model relating ecotype, acclimation temperature based on the six-days mean of daily minimum leaf temperature, and mountain range to freezing resistance, expressed as LT_50_. The alpine ecotype and the Tatra mountains were used as baseline levels. Values in bold indicate significant (<0.05) marginal effects. Conditional (R^2^_LMMm_) and marginal R² (R^2^_LMMc_) of the model were 0.69 and 0.82 respectively.

|  | Coefficient ± SE | t value | p-value |
| --- | --- | --- | --- |
| Foothill ecotype | 2.782 ± 0.651 | 4.247 | **0.005** |
| Acclimation Temperature | 0.698 ± 0.006 | 105.557 | **<0.001** |
| Niedere Tauern | 1.137 ± 0.654 | 1.739 | 0.133 |
| Făgăraș | 2.450 ± 0.654 | 3.746 | **0.010** |
| Foothill ecotype: Acclimation Temperature | -0.137 ± 0.008 | -17.095 | **<0.001** |
| Foothill ecotype: Niedere Tauern | -0.131 ± 0.925 | -1.415 | 0.207 |
| Foothill ecotype: Făgăraș | -2.890 ± 0.925 | -3.124 | **0.020** |

**Supplementary Table 5.** **Freezing resistance of leaves of diploid and tetraploid populations of *Arabidopsis arenosa* originating from the Tatra mountains.** Fixed effect coefficients of a linear mixed model relating ecotype, acclimation temperature based on the six day mean of daily minimum leaf temperature, and ploidy to freezing resistance, expressed as LT_50_. The alpine ecotype and the diploid ploidy level were used as baseline levels. Values in bold indicate significant (<0.05) marginal effects. Conditional (R^2^_LMMm_) and marginal R² (R^2^_LMMc_) of the model were 0.76 and 0.84 respectively.

|  | Coefficient ± SE | t value | p-value |
| --- | --- | --- | --- |
| Foothill ecotype | -0.591 ± 0.753 | -0.785 | 0.476 |
| Tetraploid | -1.401 ± 0.754 | -1.857 | 0.136 |
| Acclimation Temperature | 0.777 ± 0.008 | 95.797 | **<0.001** |
| Ecotype Foothill: Tetraploid | 2.717 ± 1.064 | 2.553 | 0.063 |
| Tetraploid: Acclimation Temperature | -0.112 ± 0.010 | -11.408 | **<0.001** |
